# Primary success of electrical cardioversion for new-onset atrial fibrillation and its association with clinical course in non-cardiac critically ill patients: sub-analysis of a multicenter observational study

**DOI:** 10.1186/s40560-021-00562-8

**Published:** 2021-07-08

**Authors:** Nozomu Shima, Kyohei Miyamoto, Seiya Kato, Takuo Yoshida, Shigehiko Uchino, on behalf of the AFTER-ICU study group, Tomonao Yoshida, Hiroshi Nashiki, Hajime Suzuki, Hiroshi Takahashi, Yuki Kishihara, Shinya Nagasaki, Tomoya Okazaki, Shinshu Katayama, Masaaki Sakuraya, Takayuki Ogura, Satoki Inoue, Masatoshi Uchida, Yuka Osaki, Akira Kuriyama, Hiromasa Irie, Michihito Kyo, Junichi Saito, Izumi Nakayama, Takahiro Masuda, Yasuyuki Tsujita, Masatoshi Okumura, Haruka Inoue, Yoshitaka Aoki, Takashiro Kondo, Isao Nagata, Takashi Igarashi, Nobuyuki Saito, Masato Nakasone

**Affiliations:** 1 Department of Emergency and Critical Care Medicine, Wakayama Medical University, 811-1, Kimiidera, Wakayama, Japan; 2 Intensive Care Unit, Department of Anesthesiology, Jikei University School of Medicine, Tokyo, Japan; 3 Present address: Department of Intensive Care Medicine, Tokyo Women’s Medical University, Tokyo, Japan

**Keywords:** New-onset atrial fibrillation, Electrical cardioversion, Rhythm control strategy, Mortality

## Abstract

**Background:**

Electrical cardioversion (ECV) is widely used to restore sinus rhythm in critically ill adult patients with atrial fibrillation, although its prognostic value is uncertain. This study aims to elucidate the clinical meaning of successful ECV.

**Methods:**

This is a sub-analysis of the AFTER-ICU study, a multicenter prospective study with a cohort of 423 adult non-cardiac patients with new-onset atrial fibrillation (AF). Patients that underwent ECV within 7 days after initial onset of AF were included in the sub-analysis. We compared intensive care unit (ICU) and overall hospital mortality, survival time within 30 days, cardiac rhythm at ICU discharge, and the length of ICU and overall hospital stay between patients whose sinus rhythm was restored immediately after the first ECV session (primary success group) and those in whom it was not restored (unsuccessful group). To find the factors related to the primary success of ECV, we also compared patient characteristics, the delivered energy, and pretreatment.

**Results:**

Sixty-five patients received ECV and were included in this study. Although 35 patients (54%) had primary success, recurrence of AF occurred in 24 of these patients (69%). At ICU discharge, three patients still had AF in the unsuccessful group, but no patients in the primary success group still had AF. ICU mortality was 34% in the primary success group and 17% in the unsuccessful group (*P* = 0.10). Survival time within 30 days did not differ between the groups. Delivered energy and pretreatment were not associated with primary success of ECV.

**Conclusions:**

The primary success rate of ECV for new-onset AF in adult non-cardiac ICU population was low, and even if it succeeded, the subsequent recurrence rate was high. Primary success of ECV did not affect the rate of mortality. Pretreatment and delivered energy were not associated with the primary success of ECV.

**Trial registration:**

UMIN clinical trial registry, the Japanese clinical trial registry (registration number: UMIN000026401, March 31, 2017).

**Supplementary Information:**

The online version contains supplementary material available at 10.1186/s40560-021-00562-8.

## Background

New-onset atrial fibrillation (AF) affects a substantial proportion of critically ill patients. Notably, in individuals with sepsis, a quarter of patients have complication of new-onset AF, which is associated with longer intensive care unit (ICU) stay and higher mortality [1]. Furthermore, a recent prospective study reported that longer duration of AF is associated with higher rate of in-hospital mortality among critically ill patients with new-onset AF [2]. However, therapeutic strategy for new-onset AF in critically ill patients has not been established (i.e., the rhythm control strategy or rate control strategy). In a recent randomized study that enrolled patients after cardiac surgery, rhythm control strategy for new-onset AF compared with rate control strategy did not improve the mortality rate or length of hospital stay, but it provided slightly higher percentage of maintained sinus rhythm at 60 days [3].

Electrical cardioversion (ECV) is one of the most widely used methods in the rhythm control strategy [4]. Compared with pharmacological cardioversion, it has an advantage regarding immediate restoration of sinus rhythm, and is thus usually performed in hemodynamically unstable patients. In critically ill patients, however, ECV often requires the use of additional sedatives, and the primary success rate of ECV is relatively low (35-71%). Even after primary success, the subsequent recurrence rate of AF can be as high as 46-77% [5–7].

If ECV has any clinical benefit in critically ill patients (e.g., improved mortality), it would come from restoration of sinus rhythm. Elucidation of the clinical significance of the primary success of ECV could be the first step in assessing the prognostic impact of performing ECV. In critical care literature, however, studies describing the clinical significance of the primary success of ECV are scarce. This study was therefore conducted to examine the differences between patients with and without primary success after ECV in patient characteristics, factors associated with ECV (e.g., pretreatment with pre-procedural drugs, delivered energy), and clinical outcomes. We aimed to elucidate the clinical meaning of ECV with primary success.

## Methods

This is a sub-analysis study using a cohort of Atrial Fibrillation Treatment Evaluation Registry in the ICU (AFTER-ICU) study [2, 8]. The AFTER-ICU study is a prospective cohort study conducted across 32 ICUs in Japan. It enrolled 423 adult non-cardiac patients with new-onset AF between April 1, 2017, and March 31, 2018, to examine the clinical course after the identification of new-onset AF. In summary, almost all patients that developed new-onset AF without spontaneous restoration of sinus rhythm were treated by pharmacological interventions, and anticoagulants were given to approximately 40% of the study patients. Overall hospital mortality was 26% and the incidence of in-hospital stroke was 4.5%. Detailed inclusion and exclusion criteria, methods, and results are reported elsewhere [8]. The study protocol was registered at UMIN Clinical Trial Registry (UMIN000026401) and approved by the Jikei University Institutional Review Board (28–200[8443]) and the ethics committees of all other participating hospitals with a patient or proxy opt-out policy.

In this sub-analysis, we included patients that underwent ECV within 7 days after initial AF onset or before ICU discharge if that occurred within 7 days. The AFTER-ICU study was an observational study without predefined criteria for intervention including electrical cardioversion. The decision to perform ECV was therefore made by attending physicians. We obtained the following information from the database of the AFTER-ICU study: patient characteristics, physiological data, antiarrhythmic agent usage (amiodarone, aprindine, pilsicainide, and magnesium sulfate), rate control agent usage (beta blockers and calcium channel blockers), anticoagulant usage, the timing, delivered energy and waveform of ECV, cardiac rhythm 30 s after ECV, bleeding events requiring intervention, ischemic stroke, cardiac rhythm at ICU discharge, ICU and overall hospital mortality, and the length of ICU and hospital stay.

We defined one ECV session as repeated shocks within 15 min from the first shock. Primary success was defined as restoration of sinus rhythm 30 s after the first ECV session. When evaluating each shock separately during the first ECV session, we also defined successful shock as restoration of sinus rhythm within 30 s after the shock. If antiarrhythmic agents or rate control agents were used within 6 h before the first ECV session, we considered the patient to have received pretreatment.

Continuous variables are presented as median and interquartile range, and categorical variables are presented as number and percentage (%). To find the factors related to the primary success of ECV, we compared the patient characteristics, physiological data, and pretreatment between the patients with primary success (primary success group) and those without (unsuccessful group). Furthermore, we evaluated the association between each delivered energy and successful shocks within the first ECV session. We described how cardioversion was performed and the number of shocks and delivered energy in the first ECV session. To assess whether the successful ECV affects clinical outcomes, we compared ICU and overall hospital mortality, cardiac rhythm at ICU discharge, and the length of ICU and hospital stay between the primary success group and the unsuccessful group. Ischemic stroke and bleeding events were also evaluated as adverse events. Comparisons were conducted using Pearson’s chi-square test or Fisher’s exact test for categorical variables and Mann-Whitney U test for continuous variables, as appropriate. We also used Kaplan-Meier curve and conducted log-rank tests to assess survival time within 30 days from initial AF onset.

## Results

### Demographics and characteristics

Enrolled in the AFTER-ICU study were 423 patients, 65 of whom underwent ECV and were enrolled in this sub-study. Patients that underwent ECV were younger and had less history of hypertension, but were more severely ill (e.g., higher Acute Physiology and Chronic Health Evaluation II and Sequential Organ Failure Assessment scores) than those that did not undergo ECV (Supplemental Table S1). Up to six sessions of ECV were performed (Supplemental Figures S1, S2 and S3). A total of 168 shocks were delivered to the patients in the study. All shocks were applied in biphasic waveform, except one shock in a patient without primary success of ECV, in which monophasic waveform was applied.

Thirty-five patients (54%) had primary success (primary success group). Patient demographics and clinical characteristics are shown in Table 1. Mechanical ventilation at onset of AF was received by fewer patients in the primary success group than in the unsuccessful group. Although not statistically significant, beta blocker usage at AF onset was more common in the primary success group.

**Table 1:** Patient characteristics

Variable	Primary success group (*n* = 35)	Unsuccessful group (*n* = 30)	*P* value
Age (year)	70 (61-78)	69 (65-81)	0.99
Male	24 (69)	23 (77)	0.47
APACHE II score at ICU admission	27 (22-33)	28 (21-34)	0.93
Comorbidity			
Hypertension	13 (37)	10 (33)	0.75
Diabetes	10 (29)	5 (17)	0.26
Congestive heart failure	3 (9)	1 (3)	0.62
Ischemic heart disease	3 (9)	2 (7)	1.00
Stroke or TIA	6 (17)	3 (10)	0.49
Chronic hemodialysis	2 (6)	2 (7)	1.00
Previous medication			
Antiarrhythmic agents	0 (0)	0 (0)	n/a
Beta blockers	2 (6)	2 (7)	0.87
Calcium channel blockers	7 (20)	5 (17)	0.76
Patient category			0.57
Non-scheduled surgical	5 (14)	4 (13)	
Scheduled surgical	1 (3)	3 (10)	
Medical	29 (83)	23 (77)	
Primary organ failure			0.17
Respiratory	13 (37)	12 (40)	
Gastrointestinal	6 (17)	7 (23)	
Cardiovascular	4 (11)	3 (10)	
Musculoskeletal	1 (3)	4 (13)	
Metabolic	3 (9)	0 (0)	
Hematological	3 (9)	0 (0)	
Neurological	1 (3)	1 (3)	
Trauma	1 (3)	1 (3)	
Urogenital	0 (0)	2 (7)	
Others	3 (9)	0 (0)	
**At AF onset**			
SOFA score ^a^	9 (7-14)	9 (8-13)	0.82
RRT	12 (34)	12 (40)	0.63
MV	26 (74)	28 (93)	0.04
Sedatives	21 (60)	21 (70)	0.40
Inotropes and/or vasopressors	21 (60)	21 (70)	0.40
Beta-blockers	5 (14)	0 (0)	0.06
Other antiarrhythmic agents	0 (0)	0 (0)	n/a
Infection	28 (80)	23 (77)	0.74
HR (bpm)	148 (133-173)	151 (126-173)	0.95
MAP (mmHg)	72 (61-85)	65 (59-80)	0.17

Values are given as median (interquartile range) or number (%)

Primary organ failure is based on surgical site for surgical patients or primary disease related to ICU admission for medical patients

*ECV* Electrical cardioversion, *APACHE II* Acute Physiology and Chronic Health Evaluation II, *TIA* Transient ischemic attack, *AF* Atrial fibrillation, *SOFA* Sequential Organ Failure Assessment, *RRT* Renal replacement therapy, *MV* Mechanical ventilation, *HR* Heart rate, *MAP* Mean arterial pressure, *ICU* Intensive care unit

^a^One missing data in primary success group

### Details of the first ECV session

Characteristics of the first ECV session are shown in Table 2. The number of shocks in the first ECV session was significantly lower in the primary success group. Pretreatment was received by 22 patients (63%) in the primary success group and 17 (57%) patients in unsuccessful group (*P* = 0.61). Patient characteristics including hemodynamic index did not differ between patients that received pretreatment and those that did not (Supplemental Table S2). Although not statistically significant, pretreatment with amiodarone was more common in the primary success group. There were no significant differences in the drug choice for pretreatment.

**Table 2:** Characteristics of the first ECV session

Variable	Overall (*n* = 65)	Primary success group (*n* = 35)	Unsuccessful group (*n* = 30)	*P* value
Number of shocks during the first session	1 (1-2)	1 (1-2)	2 (1-2)	0.01
Pretreatment	39 (60)	22 (63)	17 (57)	0.61
Landiolol	28 (43)	13 (37)	15 (50)	0.30
Other beta blockers	2 (3)	2 (6)	0 (0)	0.50
Amiodarone	5 (8)	5 (14)	0 (0)	0.06
Aprindine	4 (6)	3 (9)	1 (3)	0.62
Pilsicainide	4 (6)	3 (9)	1 (3)	0.62
Magnesium sulfate	5 (8)	3 (9)	2 (7)	1.00
Verapamil	3 (5)	2 (6)	1 (3)	1.00
Diltiazem	1 (2)	1 (3)	0 (0)	1.00
Anticoagulation during the first session	13 (20)	8 (23)	5 (17)	0.53

Values are given as median (interquartile range) or number (%)

*ECV* Electrical cardioversion

**Fig. 1:**
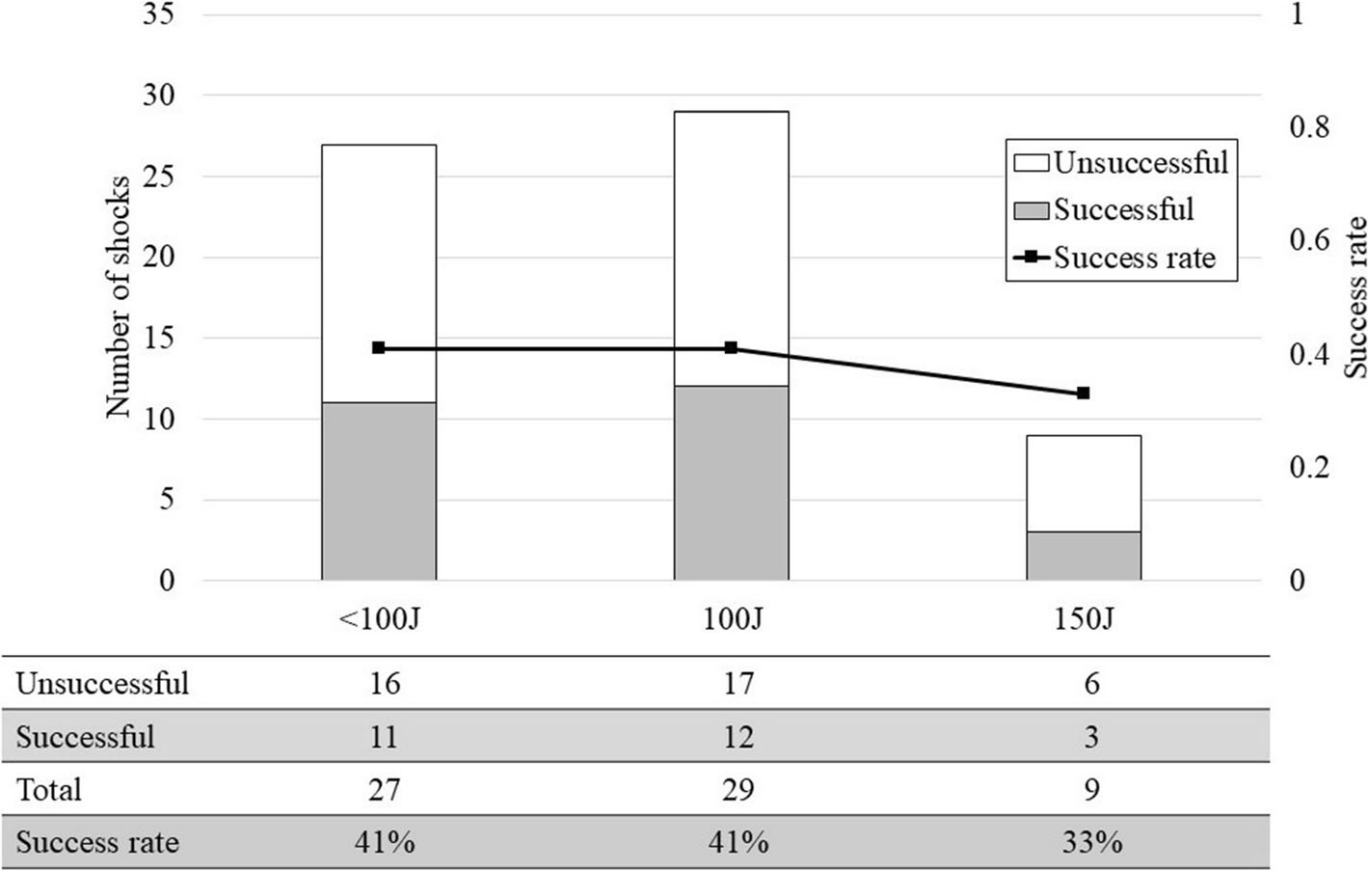
Success rate of each delivered energy in the first shock. J, joules. Gray and white bars illustrate the number of successful and unsuccessful shocks, respectively. Polygonal line shows the success rate. There was no significant association between the delivered energy and the success of electrical cardioversion (*P* = 1.00, Fisher’s exact test)

Figure 1 shows the success rate of each delivered energy in the first shock. Success rates were 41%, 41%, and 33% in patients that received < 100 Joules (J), 100 J, and 150 J, respectively. Delivered energy had no association with the success of ECV (*P* = 1.00).

**Fig. 2:**
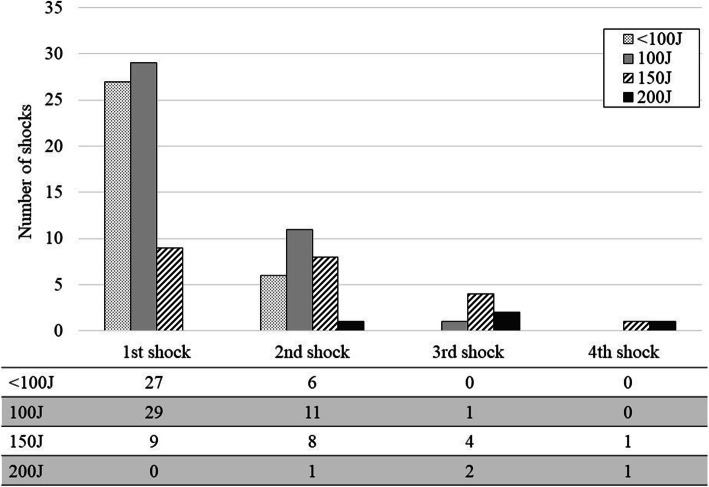
Delivered energy of each shock during the first session. J, joules. Dot pattern bars, gray bars, hatched bars, black bars show the number of shocks of less than 100 J, 100 J, 150 J, 200 J, respectively

**Fig. 3:**
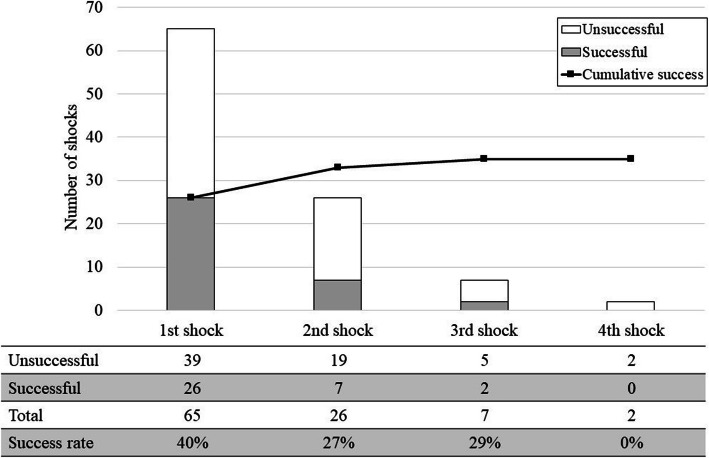
Success of each shock during the first session. A conversion to sinus rhythm within 30 s after shock was defined successful. Gray bars and white bars illustrate the number of successful and unsuccessful shocks, respectively. Polygonal line shows the number of cumulative success cases

Figure 2 shows the delivered energy of each shock during the first ECV session. In all patients, if a previous shock failed, subsequent shocks were delivered with equal to or greater energy than those in the previous shock. Figure 3 shows the success rate of each shock during the first ECV session. The success rates of the first, second, third, and fourth shocks were 40%, 27%, 29%, and 0%, respectively.

### Recurrence and outcome

In the primary success group, 14 patients had recurrence of AF within 24 h after the first session (40%), and only 11 patients (31%) maintained sinus rhythm throughout the observation period after the first ECV session. However, all survivors with primary success had sinus rhythm at ICU discharge. In contrast, among 30 patients that did not have primary success, three survivors still had AF at ICU discharge (Table 3).

**Table 3:** Outcomes and adverse events

Variable	Primary success group (*n* = 35)	Unsuccessful group (*n* = 30)	*P* value
AF at ICU discharge/survivors	0/23 (0)	3/25 (12)	0.24
ICU mortality	12 (34)	5 (17)	0.10
Length of ICU stay among survivors (day) ^a^	9 (6-13)	15 (6-24)	0.22
Hospital mortality	16 (46)	10 (33)	0.31
Length of hospital stay among survivors (day) ^b^	47 (28-64)	50 (36-103)	0.35
Anticoagulation ^c^	15 (43)	13 (43)	0.97
Bleeding event	3 (9)	1 (3)	0.62
Ischemic stroke	2 (6)	1 (3)	1.00

Values are given as median (interquartile range) or number (%)

^a^Twenty-three patients in the primary success group and 25 patients in the unsuccessful group were survived to discharge from the ICU

^b^Nineteen patients in the primary success group and 20 patients in the unsuccessful group were survived to discharge from hospital

^c^Number of patients that received anticoagulation therapy within 7 days after initial AF onset or before ICU discharge if that occurred within 7 days

*ICU* Intensive care unit

**Fig. 4:**
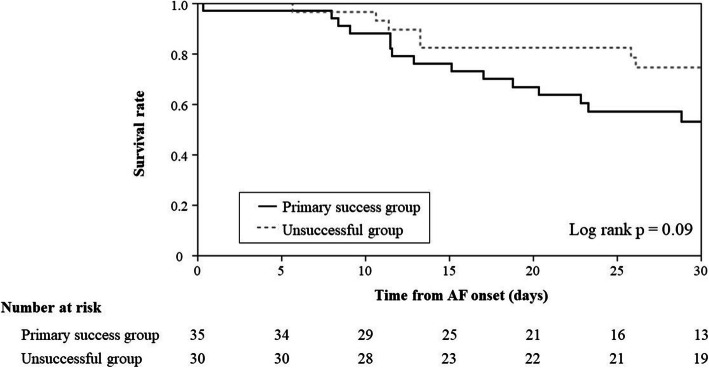
Survival rate in the primary success group and the unsuccessful group within 30 days. Black line and dotted line show the survival rate in the primary success group and the unsuccessful group, respectively. Log-rank test for comparisons of Kaplan-Meier survival curves indicated no significant difference in the survival time between groups

ICU and overall hospital mortality in the primary success group were 34% and 46%, respectively, and 17% and 33% in the unsuccessful group, with no significant difference between the groups (*P* = 0.10 and 0.31, respectively). Survival time within 30 days also did not differ between the groups (*P* = 0.09) (Fig. 4). ICU and overall hospital mortality did not significantly differ in patients with and without recurrence of AF in the primary success group. ICU mortality, 33% (8/24) for patients with recurrence vs. 36% (4/11) for patients without recurrence, *P*=1.00. Overall hospital mortality, 42% (10/24) for patients with recurrence vs. 55% (5/11) for patients without recurrence, *P*=0.72.

## Discussion

In the present study, the primary success rate of ECV was 54% (35/65 patients), and over half of the critically ill patients with new-onset AF had recurrence of AF (24/35; 69%). The delivered energy and pretreatment had no effect on the primary success of ECV. The mortality of patients with primary success of ECV did not differ from patients without primary success.

The relatively low primary success rate and high subsequent recurrence rate of ECV found in this study are concordant with those of previous studies [5–7]. Although study populations and definitions of successful ECV vary, these studies each reported an immediate ECV success rate between 35 and 71%, and the subsequent recurrence rates were between 46 and 77%. Despite the high recurrence rate of AF after the primary success, in our study, most patients were eventually discharged from ICU in sinus rhythm, which was also concordant with the previous studies [6, 7]. Various underlying mechanisms that contribute to development and continuation of AF have been suggested in recent studies (e.g., inflammation, adrenergic overstimulation, or electrolyte disturbances) [9–11]. At onset of AF, critically ill patients often coincide with these factors, which may reduce the success rate of ECV and increase the recurrence of AF. At discharge from ICU, patients have recovered from critical illness and aforementioned pathophysiology (e.g., inflammation), which might facilitate the recovery from AF and result in the high proportion of sinus rhythm at ICU discharge.

Some antiarrhythmic agents and rate control agents have been reported to improve the efficacy of ECV by increasing the success rate and preventing immediate recurrence in the non-ICU population [12–14]. In contrast, a previous study of cardiosurgical ICU patients by Arrigo et al. [7] reported that pretreatment with amiodarone did not influence the immediate success of ECV. Our study also found that pretreatment with amiodarone was not associated with the improved primary success of ECV. As for other agents, beta-blockers inhibit sympathetic overstimulation, which is one of the underlying causes of AF, as described above. However, in our study, beta-blockers did not seem to contribute to the success of ECV, suggesting that various factors other than sympathetic overstimulation may contribute to the continuation of AF in critically ill patients.

We analyzed the delivered energy used for individual shocks during the first ECV session, and found no association between the delivered energy and successful shocks. In an outpatient setting, different results have been reported; Gallagher et al. reported that high initial energy setting was more effective than low initial energy setting for long-lasting AF [15]. Also, a recent randomized controlled trial conducted outside ICU showed maximum-fixed energy ECV to be more effective than a low-escalating energy strategy [16]. Considering these results, recently published guidelines imply the superiority of maximum-fixed energy strategy over lower-escalating energy strategy [4]. This might not be the case, however, in critically ill patients, because the majority of them have multiple risk factors making them prone to AF, which may hamper the influence of the delivered energy selection on the primary success. Optimal energy of ECV is still unclear in patients in ICU, and further studies are needed to elucidate this. In the current study, escalating energy strategy was used in all patients, but the aforementioned guidelines and the randomized controlled trial upon which the guidelines were based were published after this study was conducted [4, 16]. The current choice of delivered energy was thus not a deviation from the standard practice.

ECV can provide immediate restoration of sinus rhythm compared with pharmacologic cardioversion, so patients with successful ECV have shorter periods of hemodynamic instability. In the original study of this sub-analysis, Yoshida et al. reported that sustaining duration of new-onset AF was time-dependently associated with hospital mortality [2]. We therefore hypothesized that the primary success of ECV could bring improved mortality. Contrary to our expectations, however, the present study found that patients with primary success of ECV had numerically higher mortality (ICU mortality, 34% vs. 17%, *P*=0.10; hospital mortality, 46% vs. 33%, *P*=0.31). There are several possible explanations for this. First, the recurrence rate after the primary success was as high as 69%, which means that in most cases the improvements of hemodynamics achieved by the primary success were temporary. Second, there were more bleeding events and ischemic strokes in the primary success group, although without significant difference (Table 3). Indeed, four of the five patients with primary success that had bleeding or ischemic stroke died. Thromboembolic events triggered by the primary success and subsequent recurrence of ECV might worsen the mortality rate. ECV is thus suggested to not have a large effect on mortality or maintaining of sinus rhythm. This study evaluated only the first ECV session, however, and did not fully evaluate short-term effects for hemodynamic stability. Although the short-term benefits of ECV cannot be discounted, long-term benefits (e.g., mortality) are suggested to be minimal. However, the difference may have occurred by chance owing to the small sample size. This hypothesis requires examination in further large-scale studies.

To the best of our knowledge, this is the first study to detail how ECV was performed (delivered energy, pretreatment, etc.) and how to evaluate the clinical outcomes of the primary success of ECV in critically ill patients. The numerically high mortality rate in the primary success group might be clinically relevant and should be evaluated in future studies.

Several limitations of this study should be acknowledged. First, it is a post hoc analysis using the AFTER-ICU study cohort, which aimed to elucidate the effect of AF, not that of ECV. Clinical data were therefore mainly collected at the time of AF onset, but not at the time of the first ECV. For example, in the primary success group, the use of ventilators at the onset of AF was significantly higher, but it was unclear whether the ventilators were used during the first ECV session. However, the median time from the onset of AF to the first ECV was 1.9 h and only 7 of 65 patients had ECV more than 1 day after the onset of AF. Second, the recurrence of AF may have been underestimated in patients that stayed in the ICU for more than 7 days from the AF onset, because the observation period of the AFTER-ICU study was 7 days from initial onset of AF or until the discharge from ICU if that occurred within 7 days. Indeed, 38 of 65 patients (58%) stayed in the ICU for more than 7 days after the onset of AF. However, the recurrence rate in the primary success group was high, even in the limited observation period, which does not seem to hamper the importance of our results. A third limitation is that this study did not evaluate the short-term effect of the primary success of ECV, such as the hemodynamic effect of restoring sinus rhythm. Furthermore, our study observed a greater number of patients with AF rhythm at ICU discharge in the unsuccessful group than in the primary success group. While our study cannot conclude the futility of ECV in the short term, the results suggest that these short-term effects on long-term outcomes such as mortality, if they exist, seem to be minimal.

## Conclusions

The primary success rate of ECV for new-onset AF was low in the general non-cardiac ICU population in this study. Even if ECV succeeded, the rate of subsequent recurrence was substantially high. Pretreatment and delivered energy were not associated with the success of ECV. Patients with primary success of ECV had numerically higher ICU and overall hospital mortality compared with those without success.

## Supplementary Information

**Additional file 1: Supplemental Table S1.** Characteristics and outcome of the patients that underwent electrical cardioversion and those did not. **Supplemental Table S2.** Characteristics and outcome of the patients that received pretreatment and those did not.

**Additional file 2: Supplemental Figure S1.** Success rate of each delivered energy in the first shock during each of the second to the sixth session. **Supplemental Figure S2.** Delivered energy of each shock during each of the second to the sixth session. **Supplemental Figure S3.** Success of each shock during each of the second to the sixth session.

## Data Availability

The datasets used and/or analyzed during the current study are available from the corresponding author on reasonable request.

## Abbreviations

AF: Atrial fibrillation
ECV: Electrical cardioversion
ICU: Intensive care unit
