# Supplementary material for: Primary success of electrical cardioversion for new-onset atrial fibrillation and its association with clinical course in non-cardiac critically ill patients: sub-analysis of a multicenter observational study
Source: J Intensive Care. 2021 Jul 8;9:46. doi: 10.1186/s40560-021-00562-8 (PMC8268199; doi:10.1186/s40560-021-00562-8)
Supplement: Supplementary file 1 — Additional file 1: Supplemental Table S1. Characteristics and outcome of the patients that underwent electrical cardioversion and those did not. Supplemental Table S2. Characteristics and outcome of the patients that received pretreatment and those did not. [file 40560_2021_562_MOESM1_ESM.docx]

Supplemental Table S1. Characteristics and outcome of the patients that underwent electrical cardioversion and those that did not.

| Variable | ECV group  (n = 65) | Non-ECV group  (n = 358) | *P* value |
| --- | --- | --- | --- |
| Age (yr) | 69 (63-79) | 75 (67-82) | <0.01 |
| Male | 47 (72.3) | 239 (66.8) | 0.04 |
| APACHE II score at ICU admission | 27 (22-34) | 23 (17-28) | <0.01 |
| Comorbidity |  |  |  |
| Hypertension | 23 (35) | 176 (49) | 0.04 |
| Diabetes | 15 (23) | 97 (27) | 0.50 |
| Congestive heart failure | 4 (6) | 39 (11) | 0.25 |
| Ischemic heart disease | 5 (8) | 38 (11) | 0.47 |
| Stroke or TIA | 9 (14) | 36 (10) | 0.36 |
| Chronic hemodialysis | 4 (6) | 20 (6) | 0.78 |
| Previous medication |  |  |  |
| Antiarrhythmic agents | 0 (0) | 5 (1) | 1.00 |
| Beta blockers | 4 (6) | 52 (15) | 0.06 |
| Calcium channel blockers | 12 (18) | 129 (36) | 0.01 |
| Patient category |  |  | <0.01 |
| Non-scheduled surgical | 9 (14) | 86 (24) |  |
| Scheduled surgical | 4 (6) | 57 (16) |  |
| Medical | 52 (80) | 215 (60) |  |
| Primary organ failure |  |  | 0.13 |
| Respiratory | 25 (38) | 85 (24) |  |
| Gastrointestinal | 13 (20) | 105 (29) |  |
| Cardiovascular | 7 (11) | 48 (13) |  |
| Musculoskeletal | 5 (8) | 15 (4) |  |
| Metabolic | 3 (5) | 10 (3) |  |
| Hematological | 3 (5) | 6 (2) |  |
| Neurological | 2 (3) | 22 (6) |  |
| Trauma | 2 (3) | 24 (7) |  |
| Urogenital | 2 (3) | 19 (5) |  |
| Others | 3 (5) | 24 (7) |  |
| **At AF onset** |  |  |  |
| SOFA score ^a^ | 9 (7-13) | 7 (4-10) | <0.01 |
| RRT | 24 (37) | 80 (22) | 0.01 |
| MV | 54 (83) | 201 (56) | <0.01 |
| Sedatives | 42 (65) | 139 (39) | <0.01 |
| Inotropes and/or vasopressors | 42 (65) | 167 (47) | <0.01 |
| Beta-blockers | 5 (8) | 29 (8) | 0.91 |
| Other antiarrhythmic agents | 0 (0) | 8 (2) | 0.61 |
| Infection | 51 (75) | 244 (68) | 0.10 |
| HR (bpm) | 148 (132-173) | 127 (108-145) | <0.01 |
| MAP (mmHg) | 67 (60-83) | 78 (65-90) | <0.01 |
| **Outcome** |  |  |  |
| ICU mortality | 17 (26) | 37 (10) | <0.01 |
| Hospital mortality | 26 (40) | 86 (24) | <0.01 |

Values are given as median (interquartile range) or number (%).

Primary organ failure is based on surgical site for surgical patients or primary disease related to ICU admission for medical patients.

^a^ One and ten missing data in ECV group, in non-ECV group, respectively.

ECV: electrical cardioversion, APACHE II: Acute Physiology and Chronic Health Evaluation II, TIA: transient ischemic attack, AF: atrial fibrillation, SOFA: Sequential Organ Failure Assessment, RRT: renal replacement therapy, MV: mechanical ventilation, HR: heart rate, MAP: mean arterial pressure, ICU: intensive care unit

Supplemental Table S2. Characteristics and outcome of the patients that received pretreatment and those that did not.

| Variable | Pretreatment group  (n = 39) | Non-pretreatment group  (n = 26) | *P* value |
| --- | --- | --- | --- |
| Age (yr) | 71 (66-79) | 69 (62-78) | 0.59 |
| Male | 29 (74) | 18 (69) | 0.78 |
| APACHE II score at ICU admission | 27 (23-33) | 27 (20-34) | 0.47 |
| Comorbidity |  |  |  |
| Hypertension | 16 (41) | 7 (27) | 0.30 |
| Diabetes | 9 (23) | 6 (23) | 1.00 |
| Congestive heart failure | 3 (8) | 1 (4) | 0.64 |
| Ischemic heart disease | 4 (10) | 1 (4) | 0.64 |
| Stroke or TIA | 5 (13) | 4 (15) | 1.00 |
| Chronic hemodialysis | 1 (3) | 3 (12) | 0.29 |
| Previous medication |  |  |  |
| Antiarrhythmic agents | 0 (0) | 0 (0) | n/a |
| Beta blockers | 3 (8) | 1 (4) | 0.64 |
| Calcium channel blockers | 10 (26) | 2 (8) | 0.10 |
| Patient category |  |  | 0.08 |
| Non-scheduled surgical | 3 (8) | 6 (23) |  |
| Scheduled surgical | 4 (10) | 0 (0) |  |
| Medical | 32 (82) | 20 (77) |  |
| Primary organ failure |  |  | 0.65 |
| Respiratory | 15 (38) | 10 (38) |  |
| Gastrointestinal | 6 (15) | 7 (27) |  |
| Cardiovascular | 5 (13) | 2 (8) |  |
| Musculoskeletal | 4 (10) | 1 (4) |  |
| Metabolic | 3 (8) | 0 (0) |  |
| Hematological | 1 (3) | 2 (8) |  |
| Neurological | 1 (3) | 1 (4) |  |
| Trauma | 1 (3) | 1 (4) |  |
| Urogenital | 2 (5) | 0 (0) |  |
| Others | 1 (3) | 2 (8) |  |
| **At AF onset** |  |  |  |
| SOFA score ^a^ | 9 (6-13) | 10 (8-14) | 0.22 |
| RRT | 13 (33) | 11 (42) | 0.60 |
| MV | 31 (79) | 23 (88) | 0.50 |
| Sedatives | 22 (56) | 20 (77) | 0.11 |
| Inotropes and/or vasopressors | 26 (67) | 16 (62) | 0.79 |
| Beta-blockers | 5 (13) | 0 (0) | 0.08 |
| Other antiarrhythmic agents | 0 (0) | 0 (0) | n/a |
| Infection | 28 (72) | 23 (88) | 0.13 |
| HR (bpm) | 148 (133-169) | 142 (123-179) | 0.75 |
| MAP (mmHg) | 67 (61-82) | 69 (57-91) | 0.67 |
| **Outcome** |  |  |  |
| ICU mortality | 10 (26) | 7 (27) | 1.00 |
| Hospital mortality | 15 (38) | 11 (42) | 0.80 |

Values are given as median (interquartile range) or number (%).

Primary organ failure is based on surgical site for surgical patients or primary disease related to ICU admission for medical patients.

^a^ One missing data in pretreatment group.

APACHE II: Acute Physiology and Chronic Health Evaluation II, TIA: transient ischemic attack, AF: atrial fibrillation, SOFA: Sequential Organ Failure Assessment, RRT: renal replacement therapy, MV: mechanical ventilation, HR: heart rate, MAP: mean arterial pressure, ICU: intensive care unit
