# Supplementary material for: Primary success of electrical cardioversion for new-onset atrial fibrillation and its association with clinical course in non-cardiac critically ill patients: sub-analysis of a multicenter observational study
Source: J Intensive Care. 2021 Jul 8;9:46. doi: 10.1186/s40560-021-00562-8 (PMC8268199; doi:10.1186/s40560-021-00562-8)
Supplement: Supplementary file 2 — Additional file 2: Supplemental Figure S1. Success rate of each delivered energy in the first shock during each of the second to the sixth session. Supplemental Figure S2. Delivered energy of each shock during each of the second to the sixth session. Supplemental Figure S3. Success of each shock during each of the second to the sixth session. [file 40560_2021_562_MOESM2_ESM.pdf]

Supplemental Figure S1. Success rate of each delivered energy in the first shock during each of the second to the sixth session.

Supplemental Figure S1a. 2nd session

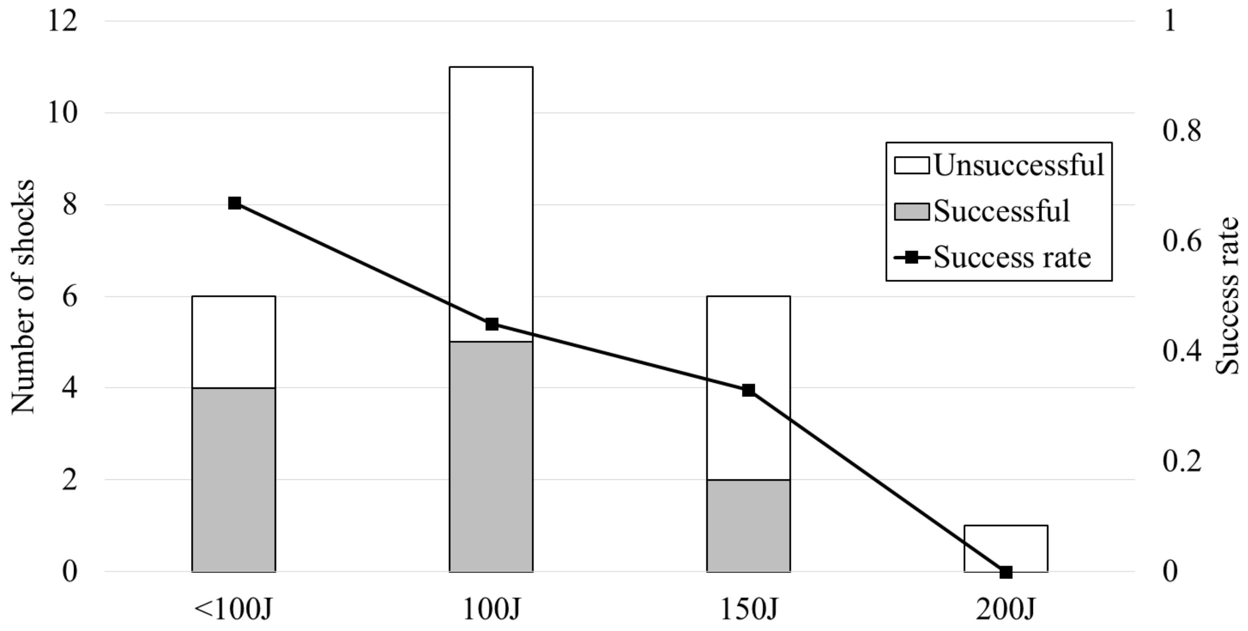

Unsuccessful	2	6	4	1
Successful	4	5	2	0
Total	6	11	6	1
Success rate	67%	45%	33%	0%

Supplemental Figure S1b. 3rd session

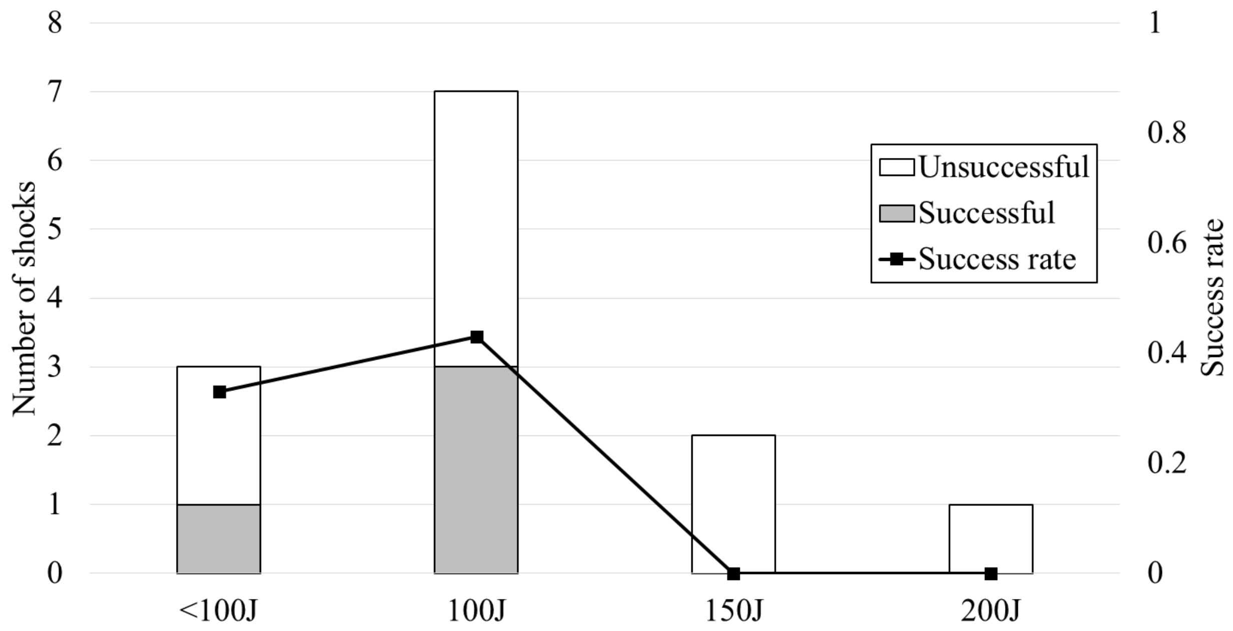

Unsuccessful	2	4	2	1
Successful	1	3	0	0
Total	3	7	2	1
Success rate	33%	43%	0%	0%

Supplemental Figure S1c. 4th session

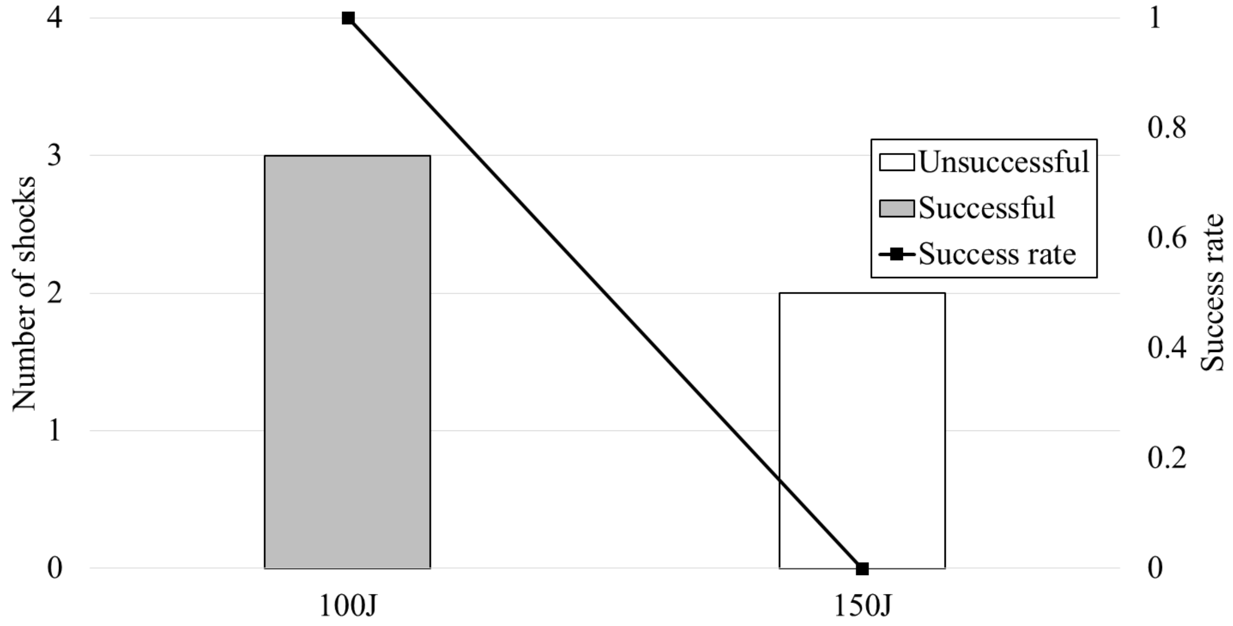

	100J	150J
Unsuccessful	0	2
Successful	3	0
Total	3	2
Success rate	100%	0%

Supplemental Figure S1d. 5th session

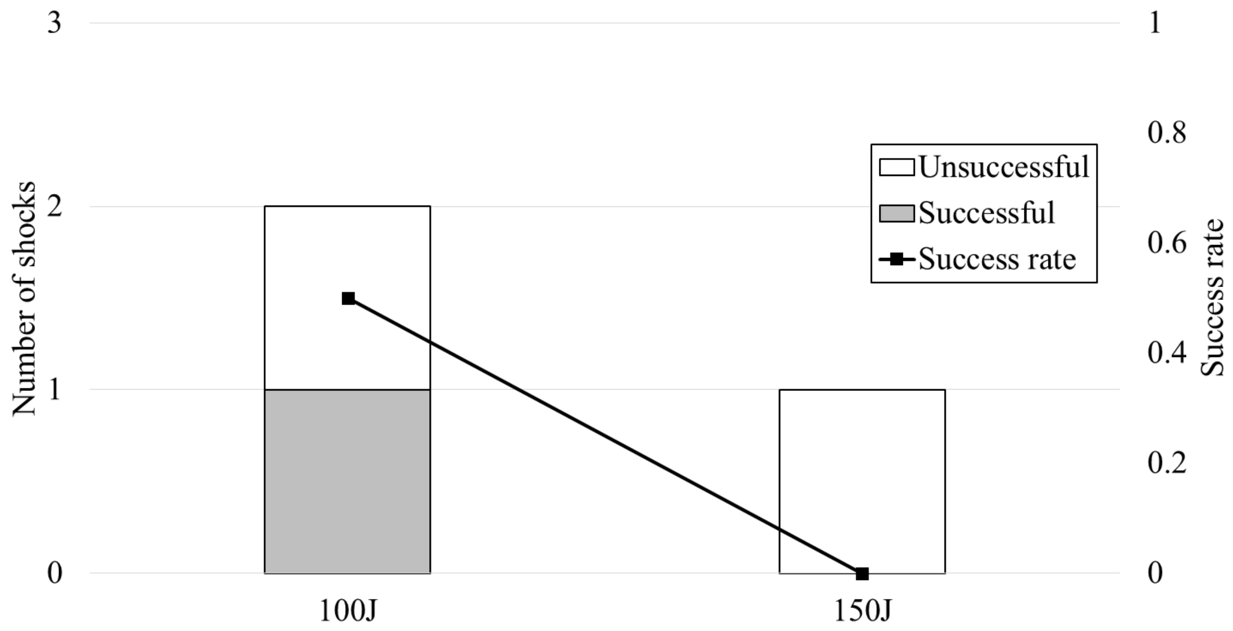

	100J	150J
Unsuccessful	1	1
Successful	1	0
Total	2	1
Success rate	50%	0%

Supplemental Figure S1e. 6th session

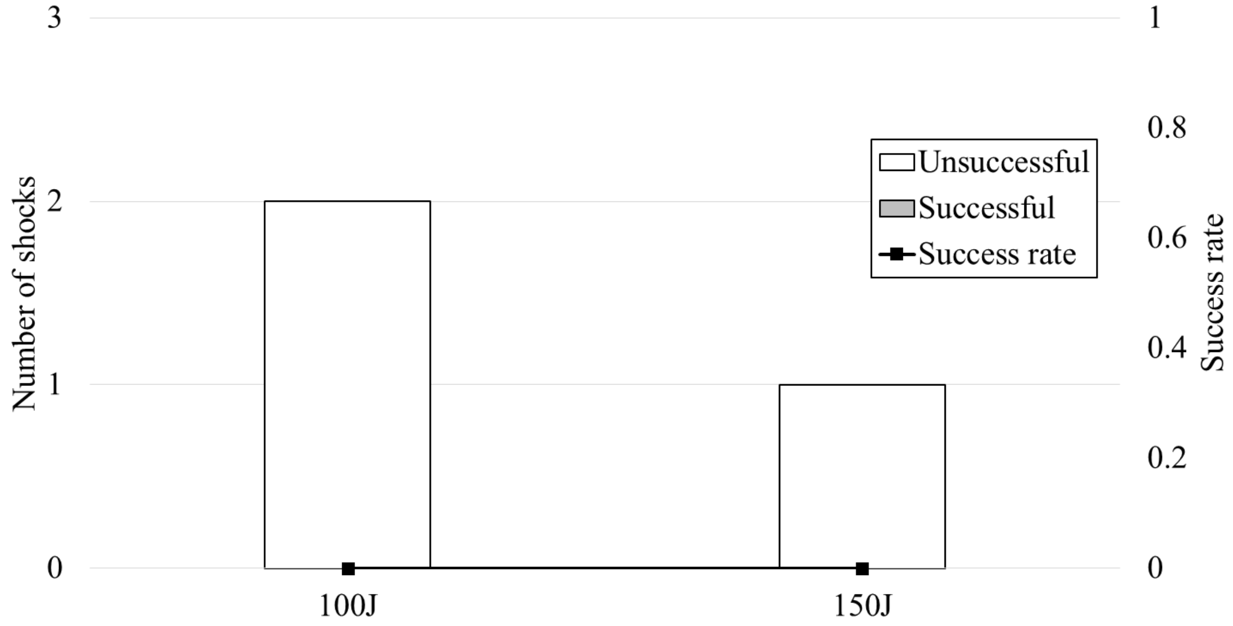

Unsuccessful	2	1
Successful	0	0
Total	2	1
Success rate	0%	0%

Supplemental Figure S2. Delivered energy of each shock during each of the second to the sixth session.

Supplemental Figure S2a. 2nd session

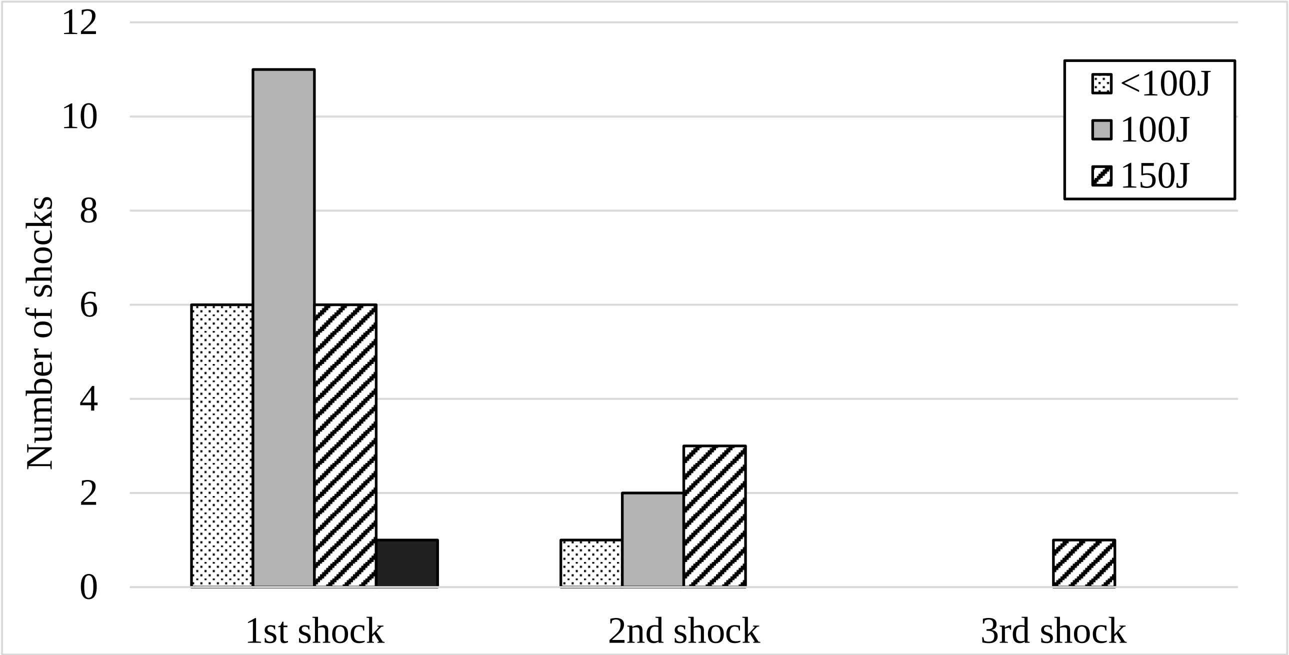

<100J	6	1	0
100J	11	2	0
150J	6	3	1
200J	1	0	0

Supplemental Figure S2b. 3rd session

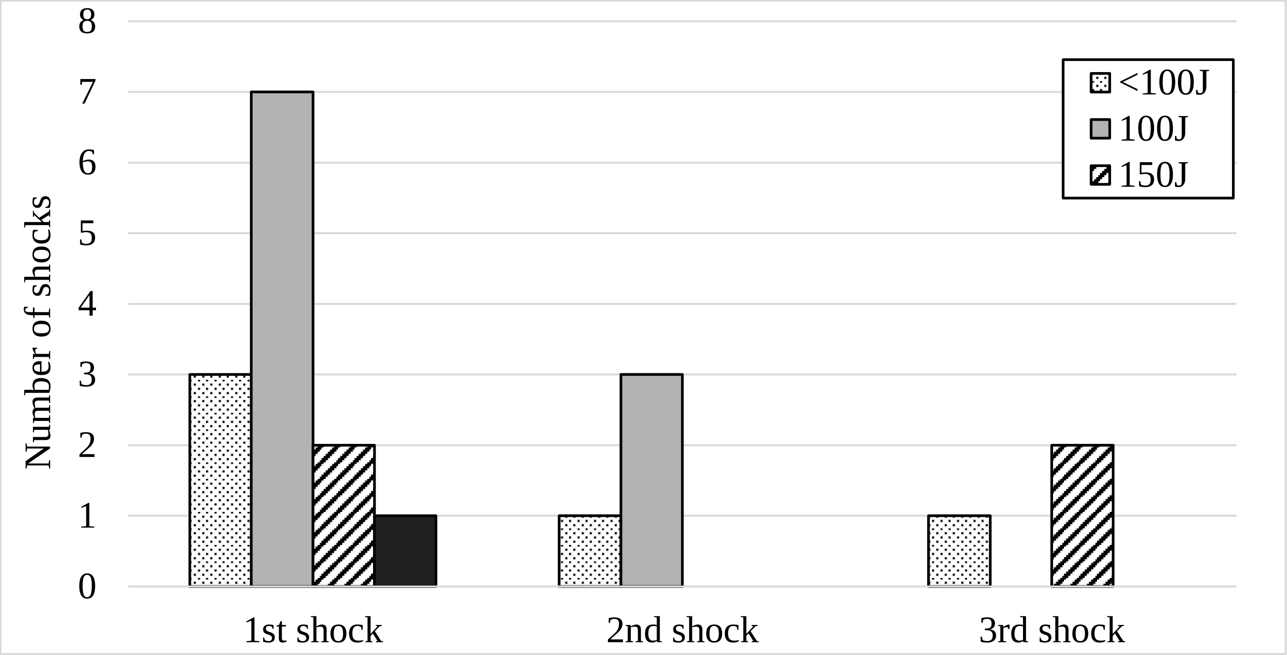

<100J	3	1	1
100J	7	3	0
150J	2	0	2
200J	1	0	0

Supplemental Figure S2c. 4th session

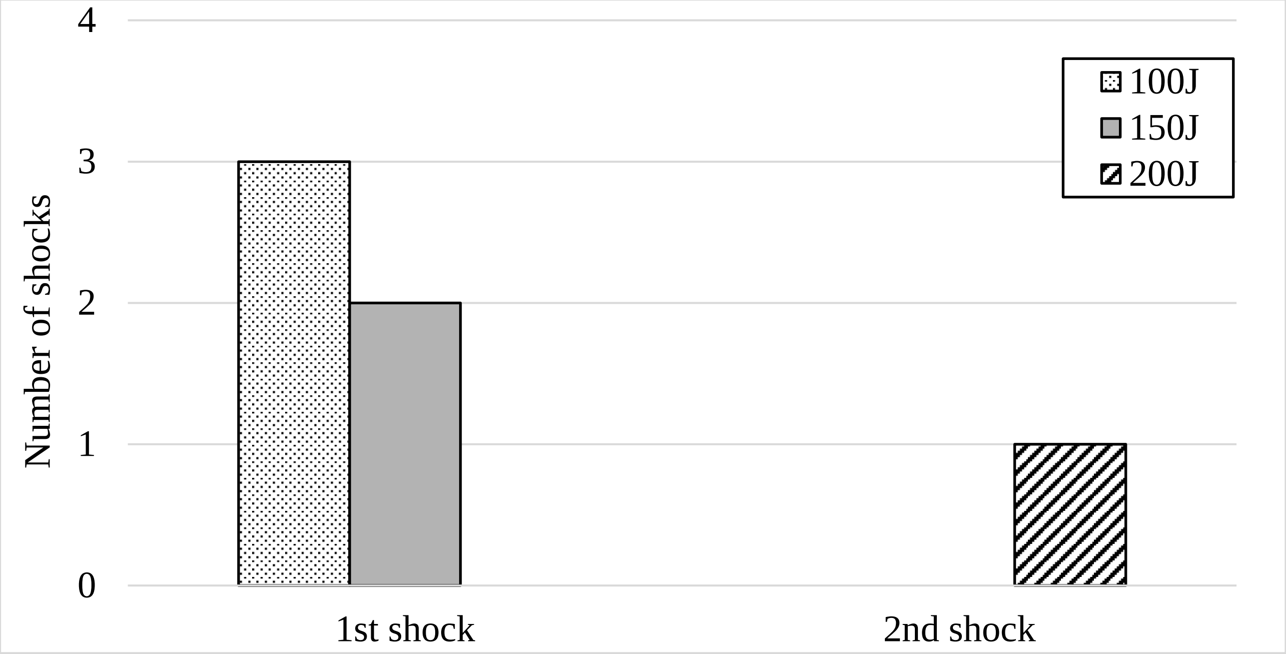

100J	3	0
150J	2	0
200J	0	1

Supplemental Figure S2d. 5th session

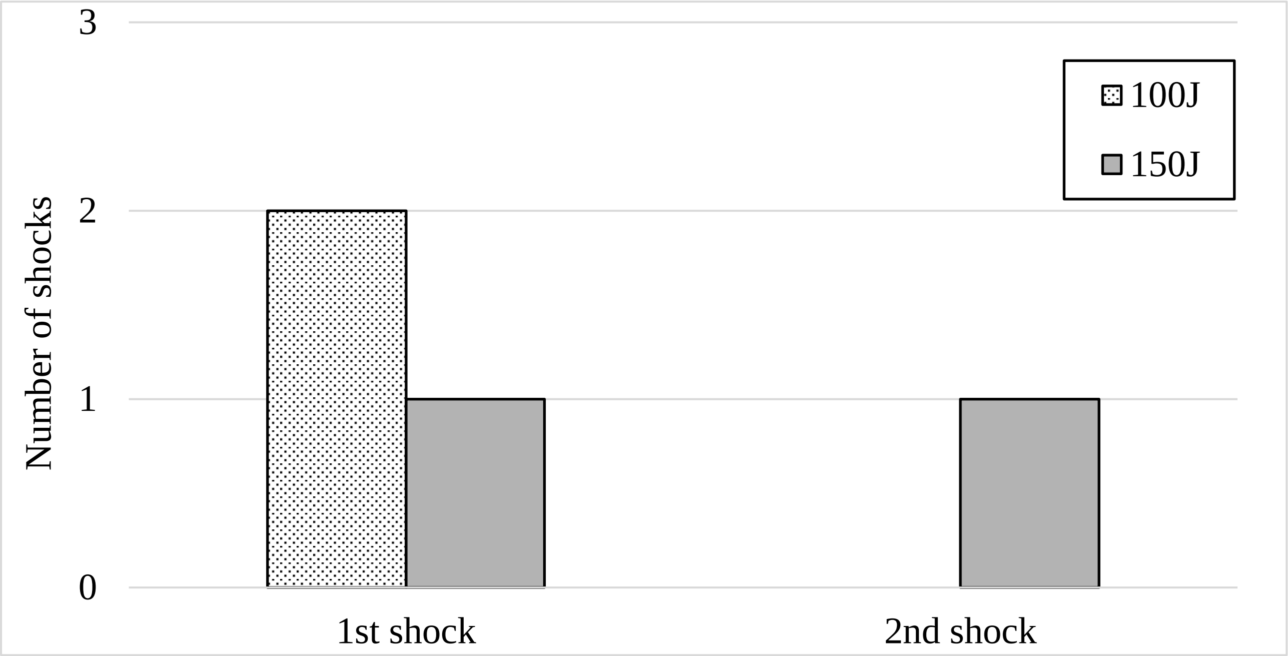

100J	2	0
150J	1	1

Supplemental Figure S2e. 6th session

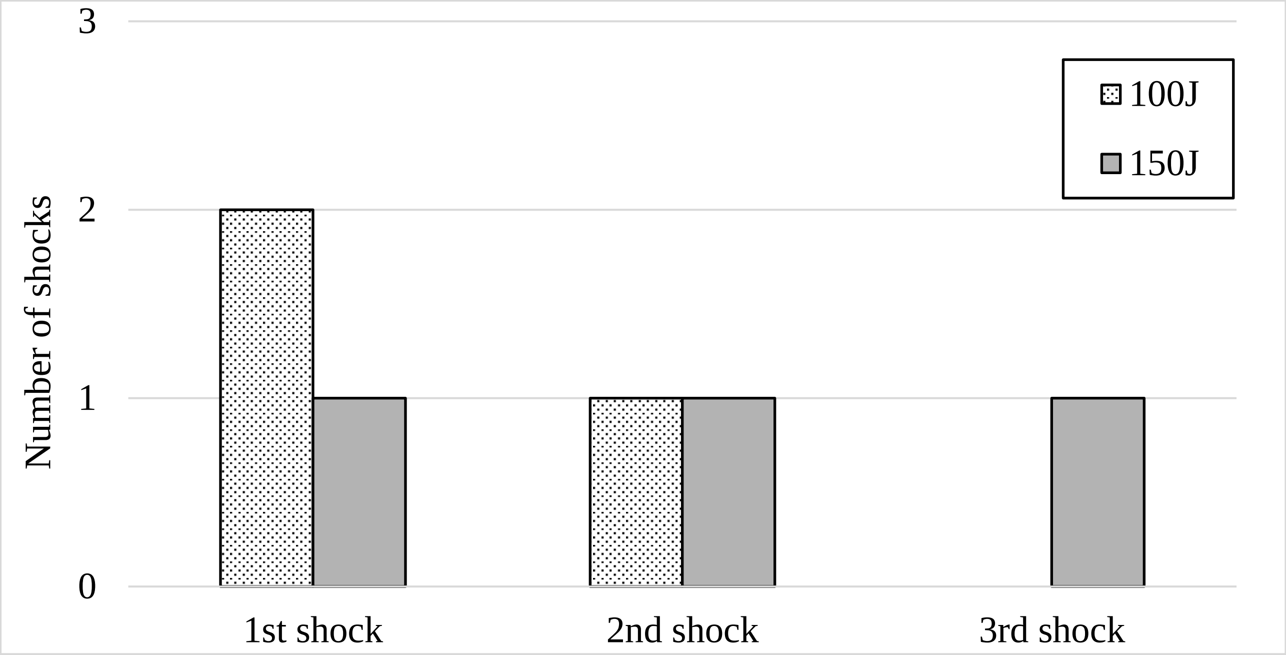

100J	2	1	0
150J	1	1	1

Supplemental Figure S3. Success of each shock during each of the second to the sixth session.

Supplemental Figure S3a. 2nd session

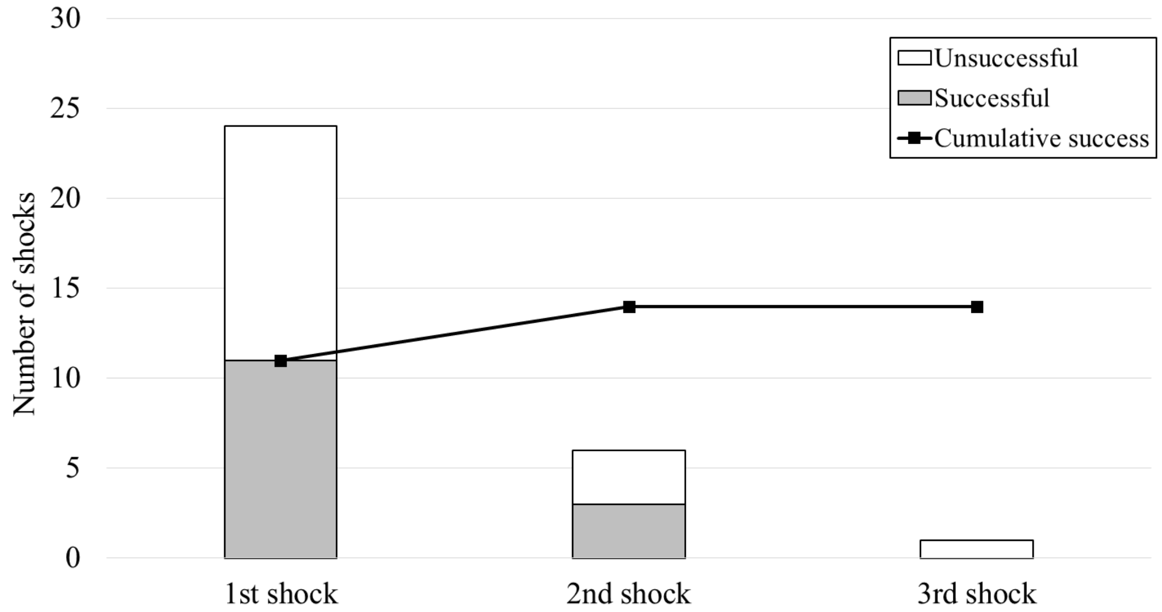

	1st shock	2nd shock	3rd shock
Unsuccessful	13	3	1
Successful	11	3	0
Total	24	6	1
Success rate	46%	50%	0%

Supplemental Figure S3b. 3rd session

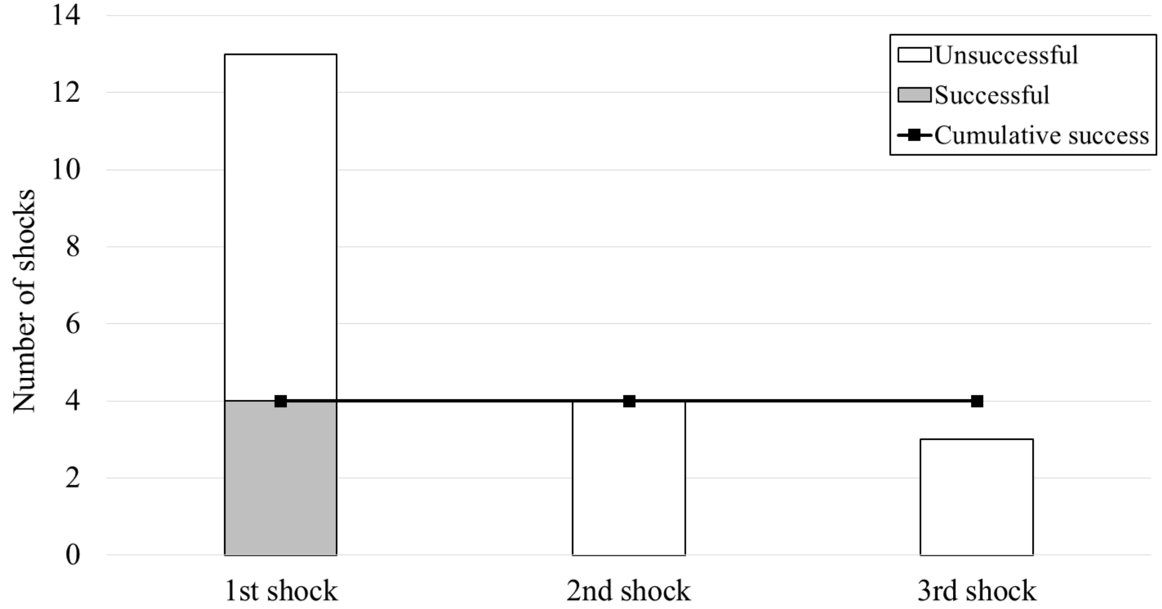

Unsuccessful	9	4	3
Successful	4	0	0
Total	13	4	3
Success rate	31%	0%	0%

Supplemental Figure S3c. 4th session

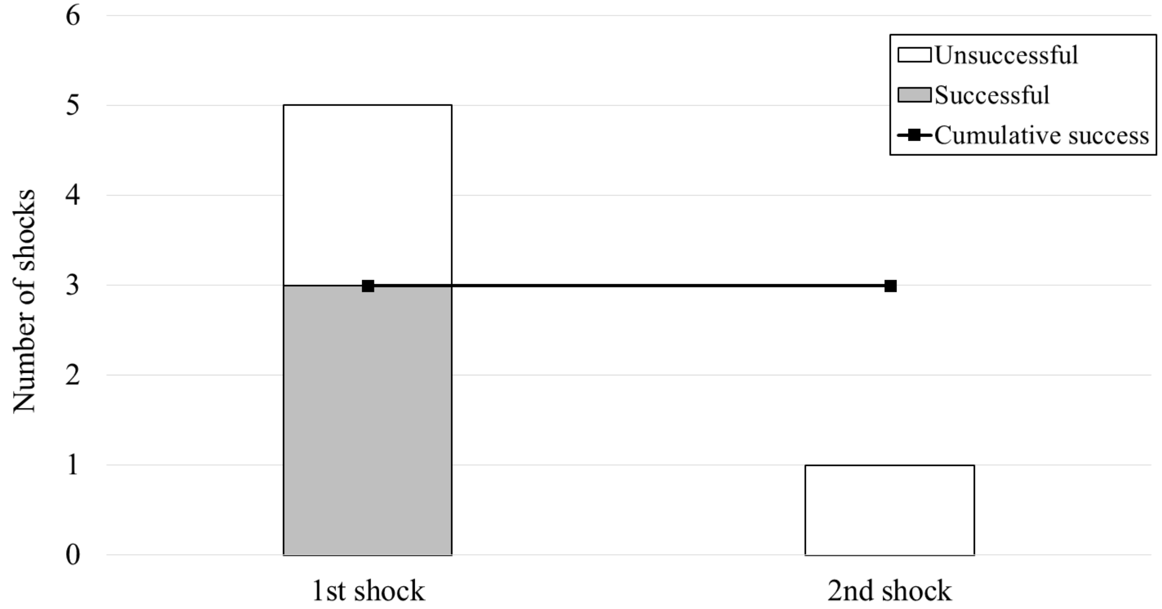

	1st shock	2nd shock
Unsuccessful	2	1
Successful	3	0
Total	5	1
Success rate	60%	0%

Supplemental Figure S3d. 5th session

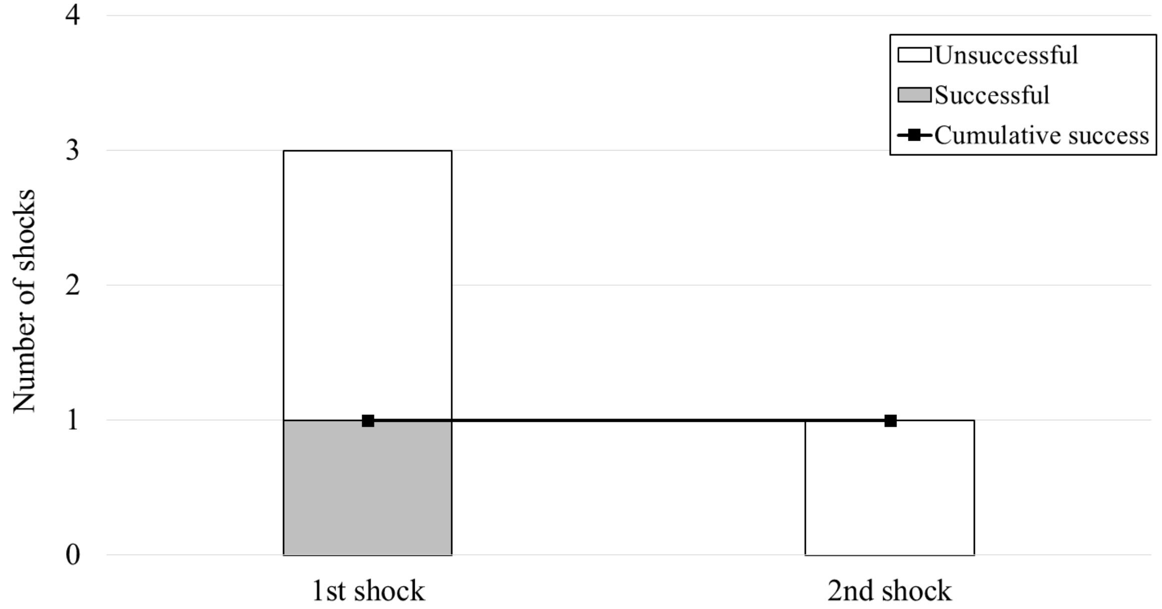

	1st shock	2nd shock
Unsuccessful	1	1
Successful	2	0
Total	3	1
Success rate	33%	0%

Supplemental Figure S3e. 6th session

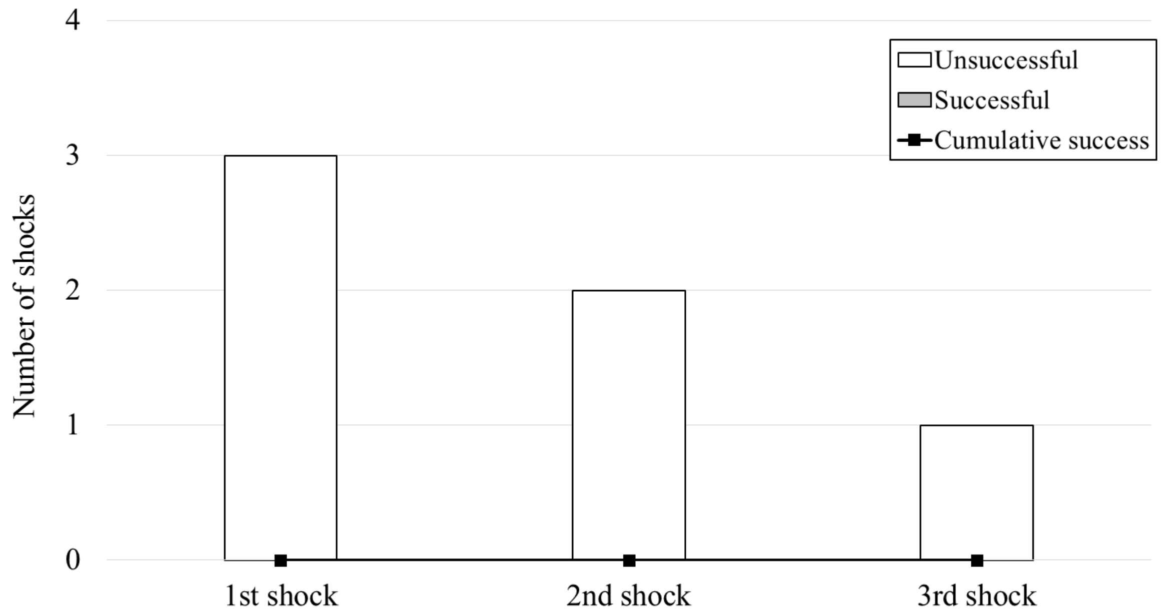

	1st shock	2nd shock	3rd shock
Unsuccessful	3	2	1
Successful	0	0	0
Total	3	2	1
Success rate	0%	0%	0%
